# Relationship of peripheral blood mononuclear cells miRNA expression and parasitic load in canine visceral leishmaniasis

**DOI:** 10.1371/journal.pone.0206876

**Published:** 2018-12-05

**Authors:** Jaqueline Poleto Bragato, Larissa Martins Melo, Gabriela Lovizutto Venturin, Gabriela Torres Rebech, Leandro Encarnação Garcia, Flavia Lombardi Lopes, Valéria Marçal Felix de Lima

**Affiliations:** 1 Department of Animal Clinic, Surgery and Reproduction, São Paulo State University (Unesp), School of Veterinary Medicine, Araçatuba, São Paulo, Brazil; 2 Department of Support, Production and Animal Health, São Paulo State University (Unesp), School of Veterinary Medicine, Araçatuba, São Paulo, Brazil; Taibah University, SAUDI ARABIA

## Abstract

Visceral leishmaniasis (VL) in humans is a chronic and often fatal disease if left untreated. Dogs appear to be the main reservoir host for *L*. *infantum* infection, however, in many regions other canids such as jackals, foxes, wolves and other mammals, such as hares or black rats, have been implicated as wild reservoirs. Most dogs cannot form an effective immune response against this infection, and this could be modulated by small non-coding RNAs, called microRNAs, responsible for post-transcriptional control of gene expression. Here, we evaluated the expression of miRNAs in peripheral blood mononuclear cells (PBMC) of symptomatic dogs naturally infected with *Leishmania (L*.*) infantum* (n = 10) and compared to those of healthy dogs (n = 5). Microarray analysis revealed that miR-21, miR-424, miR-194 and miR-451 had a 3-fold increase in expression, miR-192, miR-503, and miR-371 had a 2-fold increase in expression, whereas a 2-fold reduction in expression was observed for miR-150 and miR-574. Real-time PCR validated the differential expression of miR-21, miR-150, miR-451, miR-192, miR-194, and miR-371. Parasite load of PBMC was measured by real-time PCR and correlated to the differentially expressed miRNAs, showing a strong positive correlation with expression of miR-194, a regular positive correlation with miR-371 expression, and a moderate negative correlation with miR-150 expression in PBMC. These findings suggest that *Leishmania* infection interferes with miRNAs expression in PBMC, and their correlation with parasite load may help in the identification of therapeutic targets in Canine Visceral Leishmaniasis (CVL).

## Introduction

Visceral Leishmaniasis (VL) is a zoonosis caused by the protozoan *Leishmania infantum* and is the most fatal form of this parasitic disease [1]. Despite occurring in 76 countries, VL is still one of the most neglected diseases in the world and, of the human cases reported in America, 95.1% are in Brazil [2]. Dogs are considered the main domestic reservoirs of *L*. *infantum* [3]. Once in the vertebrate host, the parasite may cause lesions and symptoms that are characteristic of Canine Visceral Leishmaniasis (CVL), although some infected dogs may be oligo or asymptomatic [4], others may evolve to spontaneous cure [5]. The most frequent signs of VL are lymphadenopathy, onychogryphosis, cutaneous lesions, weight loss, cachexia, fever and locomotor abnormalities [6].

Protective immunity in dogs has generally been associated with a cellular immune response manifested by a positive lymphoproliferative response to *Leishmania* spp antigens [7] and cytokine production, such as IFNγ and TNF-α, which are necessary for macrophage activation and parasite death [8]. The role of T cell in the induction of the cellular response is determinant for the elimination of the parasite inside the macrophages [9].

In recent years, microRNAs (miRNAs) have been shown to play a critical role in the development and function of immune responses [10]. miRNAs are a group of small, highly conserved, single-stranded non-coding RNAs that regulate gene expression at the post-transcriptional level [11].

*In vitro* infection of human phagocytes with *Leishmania donovani* showed that the parasite induces alteration on the expression of miR-21, miR-155 and miR-146b-5p and interferes with the TGF-β signaling pathway [12]. *In vitro* infection of J774 murine macrophages with *L*. *infantum* increases miR-155, which in turn plays a role in regulating the response to *L*. *infantum* [13]. In BALB/c mice infected with *L*. *donovani*, there is a decrease in miR-122 expression, facilitating hepatic infection, and an increase in miR-122 expression decreases parasite burden in the liver [14]. *Leishmania donovani* infected mice macrophages have altered expression of miR-3620, miR-6385, miR-6973a, miR-6996, miR-328, miR-8113, miR-3473f, miR-763, miR-6540 and miR-1264, that are involved in controlling macrophage effector functions [15]. An *in silico* study has also shown that miR-29a and miR-29b target signal transcription factors that play a role in the proliferation and differentiation of T cells in visceral leishmaniasis in human, indicating that miRNAs can regulate immune response and infection control [16]. Although there are several studies on the role of miRNAs in VL, the expression of miRNAs in infected dogs, the most important reservoir for *Leishmania*, has not yet been described.

In this study, we demonstrated that there are differences in the expression of miRNAs in PBMC of dogs with VL compared to healthy dogs. The parasite load showed strong positive correlation with the miR-194 expression and regular correlation with miR-371 and miR-150 expressions in PBMC.

## Materials and methods

### Screening of animals and collection of samples

This study was approved by the Committee of Ethics in Animal Experimental Research (COBEA), with the approval of the Institutional Animal Care and Use Committee of UNESP–Universidade Estadual Paulista "Júlio de Mesquita Filho"—Araçatuba—School of Veterinary Medicine—FMVA (process 00978/2016). The generation of the dataset analysed within this manuscript is available in a data descriptor article [17].

Five healthy animals from an endemic area (Araçatuba-SP, Brazil) with absence of parasitic DNA by PCR, upon clinical examination, complete blood count and serum biochemical profile evaluation, were selected for this study, and ten animals naturally infected with *Leishmania infantum*, from the Zoonosis Control Center of Araçatuba, were selected. These animals contained at least three characteristic clinical signs of the disease, including onychogriphosis, weight loss, ear-tip injuries, periocular lesions, alopecia, skin lesions and lymphadenopathy (S1 Table). Blood samples from both groups (infected and healthy) were collected in tubes without EDTA to obtain serum for biochemical profile (S2 Table) and indirect ELISA assay (S1 Table) for the detection of anti-leishmania antibodies [18], and in EDTA tubes for complete blood count (CBC) (S3 Table) and isolation of the PBMC. Based on clinical signs, CBC results and biochemical profile, infected animals were classified in the clinical stage II of the disease [19]. PCR for detection of *Leishmania* DNA from PBMC cells was performed for all animals. Infected animals were euthanized by barbiturate anesthesia (Tiopental, Cristália Itapira, SP), followed by intravenous injection of potassium chloride 19.1% by the same route, in compliance with local laws.

### Isolation of peripheral blood mononuclear cells

PBMC were isolated by gradient Histopaque 1077 (Sigma-Aldrich) following manufacturer's instructions. They were then washed twice in phosphate buffered saline solution at pH 7.2. After isolation, these cells were counted in a Neubauer chamber for further extraction of DNA and total RNA containing the miRNAs.

### DNA extraction and determination of the *Leishmania* species

DNA extraction from PBMC samples from the experimental dogs was performed using 5x10^6^ cells with the commercial DNAeasy kit (Qiagen) according to the manufacturer's recommendations. Extracted DNA was quantified in spectrophotometer 260/280 (NanoDrop, Thermo Fisher Scientific) for evaluation of purity and concentration, and were then stored at -20°C until analysis.

Determination of the *Leishmania* species was performed by PCR-RFLP (Restriction Fragment Length Polymorphism) [20], comparing the restriction profiles of the sample with a PCR restriction profile obtained from *L*. *infantum* (IOC / L0575-MHOM / BR / 2002 / LPC-RPV), *L*. *braziliensis* (IOC / L0566-MHOM / BR / 1975 / M2903) and *L*. *amazonensis* (IOC / L0575-MHOM / BR / 1967 / PH8) as positive controls, and water as a negative control.

### Quantification of the parasite load by real-time PCR

DNA extraction from PBMC samples from the experimental dogs was performed using 5x10^6^ cells with the commercial DNAeasy kit (Qiagen) according to manufacturer's recommendation.

Parasite load quantification was performed by real-time PCR with a final reaction volume of 20μL using primers amplifying a 116bp fragment of the kinetoplast DNA (kDNA) of *Leishmania* spp. (5' CCTATTTTACACCAACCCCCAGT 3' and 5 'GGGTAGGGGCGTTCTGCGAAA 3'), at a concentration of 900nM [21], Power SYBR Green PCR Master Mix (Applied Biosystems) and 50ng of sample DNA. Amplification condition used was comprised of an initial heating of 95°C for 10 minutes, 40 cycles of 95°C for 15 seconds and 65°C for 60 seconds. Upon the end of amplification, a dissociation curve of the amplified fragment was determined from 60°C to 95°C with an increase of 0.5°C every 5 seconds. A standard curve with DNA from *Leishmania infantum* promastigotes (MHOM/BR00/MER02) with a serial dilution of 10^8^ to 10^1^ of parasite DNA was performed for each reaction.

### Extraction and quantification of miRNA

Extraction of total RNA from 5x10^6^ PBMC was performed on the samples with the commercial mirVana kit for isolation of miRNA with phenol (Life Technologies), following the procedure indicated by the manufacturer. Following isolation, total RNA was stored at -80°C until evaluation of quality and concentration.

Isolated RNAs were analyzed by spectrophotometry (NanoDrop, Thermo Fisher Scientific) for evaluation of their quantity and purity (260/280). Before performing microarray, samples were also analyzed for RNA quality by capillary electrophoresis (Bioanalyzer, Agilent Technologies) using the commercial Agilent RNA 6000 Nano kit, following manufacturer's instructions.

### Microarray

Total RNAs with satisfactory amount (over than 30ng/μl) and quality (RNA integrity number > 8) were used to perform microarray analysis using a miRNA 4.1 Array Strip (Affymetrix) containing probes designed for the miRNAs of several species, including 291 from *Canis familiaris*.

MicroRNAs were biotinylated using the Affymetrix FlashTag Biotin HSR RNA Labeling Kit following manufacturer's instructions. For the microarray, GeneAtlas Hybridization, Wash, and Stain Kit for miRNA array Strips were used, following manufacturer's instructions.

Microarray data were deposited in Gene Expression Omnibus with the access number GSE105443 according to the minimum information about microarray experiment (MIAME) standards.

### Microarray data analysis

Normalization and quality of microarray analysis of the miRNAs of control and infected dogs were performed in the Expression Console Software program, version 1.4.1 (Affymetrix, Thermo Fisher Scientific). Differential analysis of the miRNAs was performed in the Transcriptome Analysis Console (Affymetrix, Thermo Fisher Scientific).

The miRBase database (mirbase.org) was used to check the homology between the sequences of all differentially expressed non-canine miRNAs with known canine miRNAs.

Analysis of targets and pathways for the differentially expressed and validated miRNAs in dogs with VL were performed using the Ingenuity Pathway Analysis program (Qiagen).

Gene Ontology (GO) enrichment analysis was performed using the ENRICHR program (http://amp.pharm.mssm.edu/Enrichr/) [22,23].

### Real-time PCR for miRNAs analysis

To validate the results obtained by microarray, real-time PCR (qPCR) was performed. cDNA production was performed using the miScript RT II kit (Qiagen), as recommended by the manufacturer. qPCR reactions were performed using commercially available specific primers for our *Canis familiaris* miRNAs of interest and the endogenous reference SNORD96A (miScript, Qiagen) using the SYBR Green system (miScript SYBR Green PCR kit, Qiagen) in real-time thermal cycler (RealPlex, Eppendorf). Amplification conditions were determined by the manufacturer. All miRNAs (including the reference) were run in separate plates. For each miRNA, a standard curve with a serial dilution of a pool of the cDNAs was performed. Absolute quantification of each miRNA was performed by converting sample cycle threshold values to a concentration (ng/μl), based on the standard curves, which were generated using 10-fold serial dilutions of the pool of cDNAs. Target amount was then divided by SNORD96A levels to obtain a normalized target value. All samples were evaluated in duplicate.

### Statistical analysis

Statistical analysis were performed using the GraphPad Prism 6 software (GraphPad Software, Inc., La Jolla, CA, USA). Analysis of variance (ANOVA) was performed for treatment comparison in the microarray. Mann-Whitney test was performed for the miRNA qPCR results. Spearman correlation was done to evaluate the association between miRNA expression and blood count, biochemical molecules and parasite load. Fisher test was used by IPA for canonical pathway analysis. Results were considered significant when p<0.05.

## Results

### *Leishmania infantum* was identified in dogs with VL

PCR-RFLP was performed to identify the *Leishmania* species. In all dogs of the infected group, *L*. *infantum* was found as the causative agent, as shown in S1 Fig.

### Differentially expressed microRNAs and validation in PBMC of dogs with VL compared to healthy dogs

**Fig 1:**
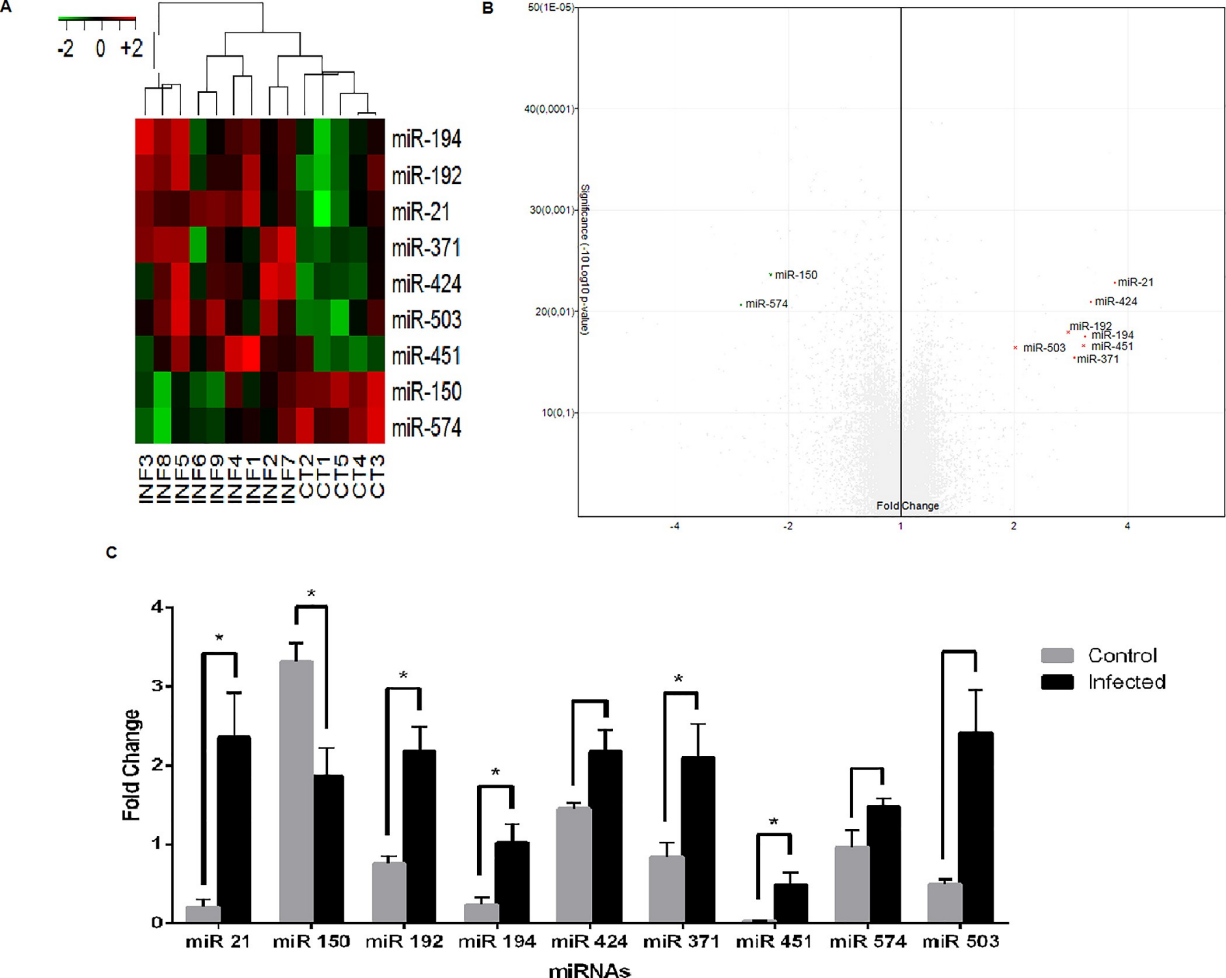
Differentially expressed miRNAs in PBMC of dogs infected with *L*. *infantum*. (A) Heatmap of differentially expressed miRNAs in PBMC of dogs infected with *L*. *infantum* compared to control group. Heatmap shows the average signal of miRNAs. Upregulated miRNAs are plotted in red and downregulated miRNAs are plotted in green. Fold change cut-off was lower than -2 and greater than 2. Analysis of variances (ANOVA) was performed between groups. (B) To the left, in green, are illustrated the miRNAs with negative expression and to the right in red the miRNAs with positive expression compared to control. miRNAs illustrated in grey hybridized, but did not show homology with known canine miRNAs. (C) miRNAs validated by real-time PCR. Expression of microRNAs in infected and healthy groups was quantified by real-time PCR in PBMC of infected and healthy dogs. Data represent the mean values of miRNA expression +/- standard error of the mean, and the asterisks represent statistically significant data following Mann-Whitney test. Results was considered significant when p<0.05.

Considering that miRNAs can regulate immune response [11], microarray was employed for comparative analysis of miRNA expression in the blood of dogs naturally infected with *L*. *infantum* and healthy dogs. miRNAs miR-194, miR-192, miR-21, miR-424, miR-451, miR-503 and miR-371 showed increased expression in the blood of infected dogs (3.1, 2.8, 3.7, 3.2, 3.1, 2.0 and 2.9 fold change respectively), while miR-150 and miR-574 showed decreased blood expression (-2.2 and -2.7 fold change respectively). S4 Table shows canine differentially expressed miRNAs in dogs with VL and respective fold change and p-value. Heatmap (Fig 1A) and Volcano plot (Fig 1B) show the differentially expressed miRNAs in PBMC of dogs with VL compared to healthy ones. miRNAs of other species (mammals, birds, fish, plants) hybridized in the microarray, but none of them showed homology with currently described canine sequences. The percentage of upregulated canine miRNAs was 2,40% (7/291) and downregulated was 0,68% (2/291).

To confirm the differential expression of miRNAs in the blood of dogs with VL in the microarray, real-time PCR validation was performed. Reactions showed efficiency from 0.84 to 1.42, slope from -2.603 to 3.761 and R^2^ from 0.933 to 0.998 (S5 Table).

miR-21, miR-451, miR-192, miR-194 and miR-371 were increased similarly to the results seen for the microarray, and miR-150 decreased in the PBMC (Fig 1C).

### Canonical pathways regulated by differentially expressed miRNAs in PBMC of infected animals

**Fig 2:**
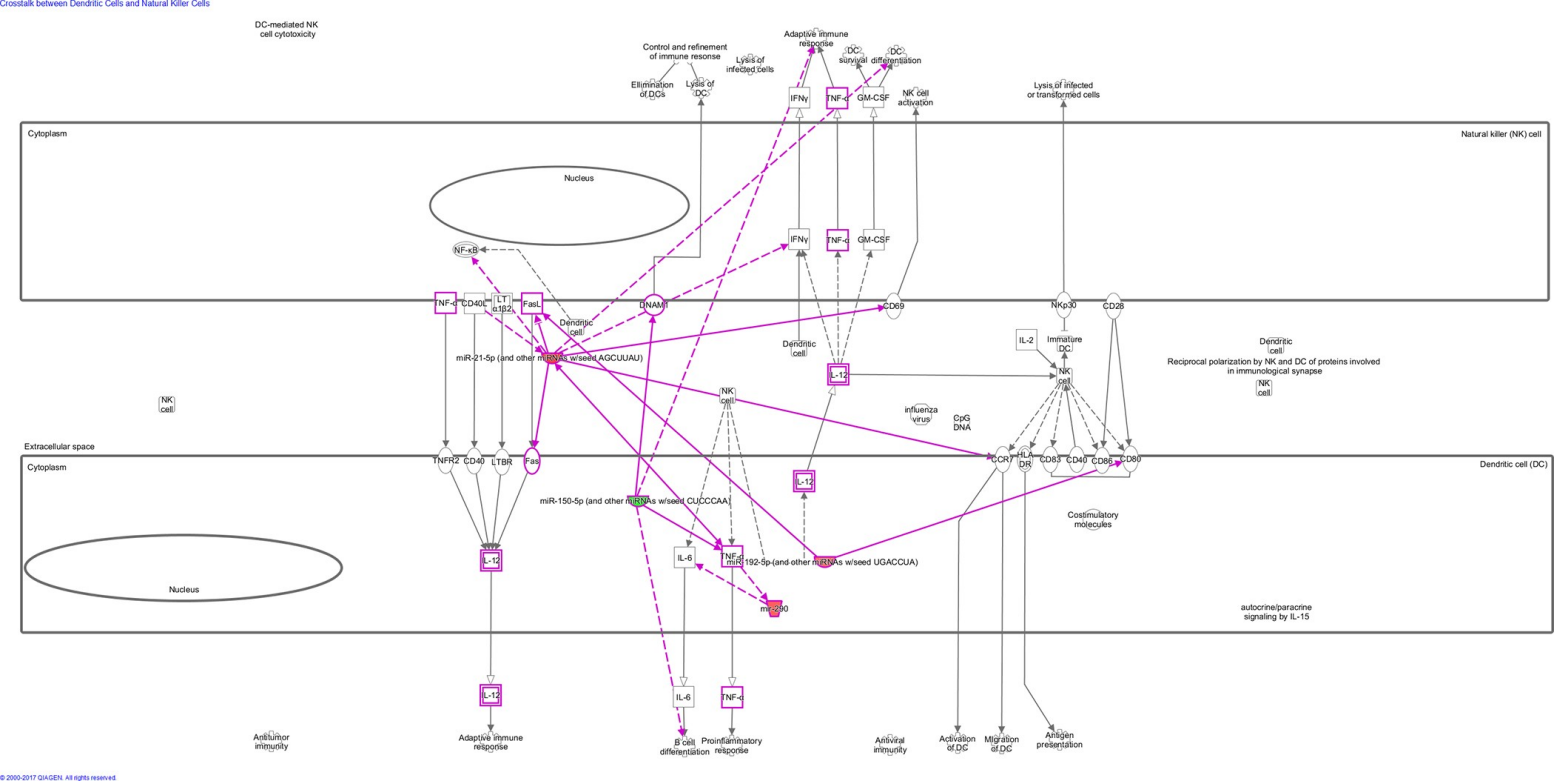
IPA’s Canonical Pathway “crosstalk between dendritic cells and natural killer cells”. Upregulated miRNAs are in red, and downregulated are in green. Indirect relationships are represented by dotted lines and direct relationships by solid lines. miR-290 is a synonym of miR-371 in IPA’s analysis. miR-192 targets CD80 and FASL. miR-371 targets IL-6 and TNF-α. miR-150 targets TNF-α and DNAM-1. miR-21 targets FAS, FASL, TNFα, CD40L, NFκB, IFNγ, CD69 and CCR7. This figure was obtained with Ingenuity Pathway Analysis (Qiagen).

IPA's microRNA Target Filter (employing highly predicted and experimentally observed only) showed target genes and demonstrated 63 canonical pathways regulated by these miRNAs/targets (S6 Table). The top 20 canonical pathways and target genes are presented in Table 1, including p53 signaling, antiproliferative role of TOB in T cell signaling, STAT3 pathway, PTEN signaling, death receptor signaling and crosstalk between dendritic cells and natural killer cells, that can regulate the immune response in CVL. We further showed the canonical pathway “crosstalk between dendritic cells and natural killer cells” (Fig 2), which targets important genes involved in immunopathogenesis in CVL, such as NFκB, TNF-α, CD80, IFN-γ and DNAM-1.

**Table 1:** Top twenty canonical pathways predicted for the differentially regulated miRNAs in CVL.

Ingenuity Canonical Pathways	P value	miRNAs in the Pathway	Target genes
Aryl Hydrocarbon Receptor Signaling	0.00014	miR-150	CDKN1B, CHEK2, HSP90B1, IL1A, TNF, TP53
		miR-192	ATM, DHFR, FASLG, GSTA2, RB1
		miR-194	ALDH4A1, GSTT2, IL1A, MAPK1
		miR-21	ALDH1A1, APAF1, CDK6, CDKN1A, FAS, FASLG, IL1B, NFIA, NFIB, TNF
p53 Signaling	0.00015	miR-150	CHEK2, MDM4, PERP, TP53
		miR-192	ATM, PERP, RB1, STAG1
		miR-194	CCNG1, CSNK1D, SERPINE2, THBS1
		miR-21	APAF1, CCNG1, CDKN1A, FAS, FRS2, PIK3R1, PTEN, SERPINB5, TNFRSF10B
Tumoricidal Function of Hepatic Natural Killer Cells	0.00030	miR-150	GZMB
		miR-192	CYCS, FASLG
		miR-194	CASP7, SRGN
		miR-21	APAF1, CASP8, FAS, FASLG
Osteoarthritis Pathway	0.00068	miR-150	CEBPB, CREB1, MMP13, TNF, VEGFA
		miR-192	ADIPOQ, FGF2, HTRA1, IL1RAP, SDC4, TGFBR1
		miR-194	CASP7, FZD6, RAC1
		miR-21	BMPR2, CASP8, FGF18, FZD6, GLIS2, IL1B, JAG1, RBPJ, SMAD7, TGFBR2, TIMP3, TNF, VEGFC
Hepatic Fibrosis / Hepatic Stellate Cell Activation	0.00098	miR-150	IL1A, MMP13, PDGFB, STAT1, TNF, VEGFA
		miR-192	CCR5, CSF1, FASLG, FGF2, FLT1, IL1RAP, SERPINE1, TGFBR1
		miR-194	CXCL3, EDN1, IL1A
		miR-21	ACTA2, CCR7, FAS, FASLG, IL1B, IL6R, KLF6, SMAD7, TGFBR2, TNF, TNFRSF11B, VEGFC
Crosstalk between Dendritic Cells and Natural Killer Cells	0.00141	miR-150	CD226, TNF
		miR-192	CD80, FASLG
		miR-194	ACTG2, CD83, FSCN3
		miR-21	ACTA2, CCR7, CD69, FAS, FASLG, IL12A, TNF
dTMP De Novo Biosynthesis	0.00182	miR-192	DHFR, DHFR2, TYMS
Cell Cycle: G1/S Checkpoint Regulation	0.00186	miR-150	CDKN1B, HDAC8, SKP1, TP53
		miR-192	ATM, RB1
		miR-194	BMI1, E2F6, HDAC8
		miR-21	CDC25A, CDK6, CDKN1A, SKP2
STAT3 Pathway	0.00347	miR-192	FLT1, PIM1, SOCS6, TGFBR1
		miR-194	MAPK1, RAC1, RRAS2, SOCS2
		miR-21	BMPR2, CDC25A, CDKN1A, MAPK10, SOCS5, SOCS6, STAT3, TGFBR2
Human Embryonic Stem Cell Pluripotency	0.00363	miR-150	FOXD3, PDGFB
		miR-192	ATM, FGF2, LEFTY2, TGFBR1
		miR-194	ACVR1, FZD6, H2BFM
		miR-21	BMPR2, FRS2, FZD6, NTF3, PIK3R1, SMAD7, SOX2, TGFBR2
Estrogen-mediated S-phase Entry	0.00417	miR-150	CDKN1B
		miR-192	RB1
		miR-194	E2F6
		miR-21	CDC25A
Actin Cytoskeleton Signaling	0.00427	miR-150	ARPC3, FGF16, PDGFB, TMSB4Y
		miR-192	ATM, BRK1, CRK, FGF14, FGF2, FGF7, MSN, MYLK, PIP4K2B
		miR-194	ACTG2, CFL2, GNA13, LIMK2, MAPK1, PFN2, PIP4K2C, RAC1, RRAS2, TMSB4Y
		miR-21	ACTA2, ARHGAP24, ARHGEF12, CFL2, FGF18, FRS2, PFN2, PIK3R1, SOS2, TIAM1, TMSB4Y
Type I Diabetes Mellitus Signaling	0.00427	miR-150	GZMB, STAT1, TNF
		miR-192	CD80, CYCS, FASLG, GAD1, HLA-DOB, IL1RAP, SOCS6
		miR-194	GAD1, MAPK1, SOCS2, TRAF6
		miR-21	APAF1, CASP8, FAS, FASLG, IL12A, IL1B, MAP2K3, MAPK10, SOCS5, SOCS6, TNF, TNFRSF11B
Cyclins and Cell Cycle Regulation	0.00437	miR-150	CDKN1B, HDAC8, SKP1, TP53
		miR-192	ATM, PPP2CB
		miR-194	E2F6, HDAC8
		miR-21	CDC25A, CDK6, CDKN1A, SKP2
Regulation of the Epithelial-Mesenchymal Transition Pathway	0.00468	miR-150	FGF16, ZEB1
		miR-192	ATM, FGF14, FGF2, FGF7, TGFBR1, ZEB1, ZEB2
		miR-194	FZD6, MAPK1, RRAS2
		miR-21	FGF18, FRS2, FZD6, JAG1, MAP2K3, PIK3R1, RBPJ, SOS2, STAT3, TGFBR2
Antiproliferative Role of TOB in T Cell Signaling	0.00525	miR-150	CDKN1B, SKP1
		miR-192	PABPC4, RB1, TGFBR1
		miR-194	MAPK1
		miR-21	SKP2, TGFBR2
Molecular Mechanisms of Cancer	0.00603	miR-150	CDKN1B, CHEK2, RAPGEF3, TP53
		miR-192	ATM, CRK, CYCS, FASLG, PRKAR1A, PRKCQ, RALB, RB1, TGFBR1, XIAP
		miR-194	ADCY7, CASP7, CDK14, E2F6, FNBP1, FZD6, GNA13, H2BFM, MAPK1, PRKAR1A, RAC1, RAP2B, RRAS2
		miR-21	APAF1, ARHGEF12, BMPR2, CASP8, CDC25A, CDK6, CDKN1A, FAS, FASLG, FRS2, FZD6, PIK3R1, SMAD7, TGFBR2
PTEN Signaling	0.00603	miR-150	CDKN1B
		miR-192	FASLG, FLT1, TGFBR1
		miR-194	H2BFM, MAPK1, RAC1, RRAS2
		miR-21	BMPR2, CDKN1A, FASLG, PIK3R1, PREX2, PTEN, SOS2, TGFBR2
Small Cell Lung Cancer Signaling	0.00631	miR-150	CDKN1B, CKS1B, TP53
		miR-192	ATM, CYCS, RB1, TRAF5
		miR-194	CKS1B, MAPK1, RRAS2, TRAF6
		miR-21	APAF1, CDK6, FRS2, PIK3R1, PTEN, SKP2, SOS2
Death Receptor Signaling	0.00912	miR-150	MAP4K4, TNF
		miR-192	CYCS, FASLG
		miR-194	XIAP, ACTG2, CASP7, MAP4K4, PARP11
		miR-21	ACTA2, APAF1, CASP8, FAS, FASLG, TNF

To understand the functional networks of the target genes, GO analysis was performed. GO analysis showed that these target genes were involved in a large number of physiological processes at the levels of biological processes, cellular component and molecular function (S7–S9 Tables).

### Blood count, biochemical molecules and correlation with the expression of microRNAs miR-194 and miR-371 in dogs naturally infected with *L*. *infantum*

There was a strong positive correlation between miR-194 expression and serum urea (r = 0.73 p = 0.031) (S2A Fig). There was a strong negative correlation between miR-194 expression and hemoglobin concentration (r = -0.70 p = 0.037) (S2B Fig) and a strong negative correlation between miR-371 expression and globular volume (S2C Fig) (r = -0.76 p = 0.019).

### Parasite load on PBMC and correlation with expression of microRNAs miR-194, miR-371 and miR-150 in dogs naturally infected with *L*. *infantum*

Real-time PCR reaction for quantification of parasite load presented efficiency values of 0.97, slope -3.405 and R^2^ 0.958. Parasite load ranged from 10.1ng to 28633ng.

**Fig 3:**
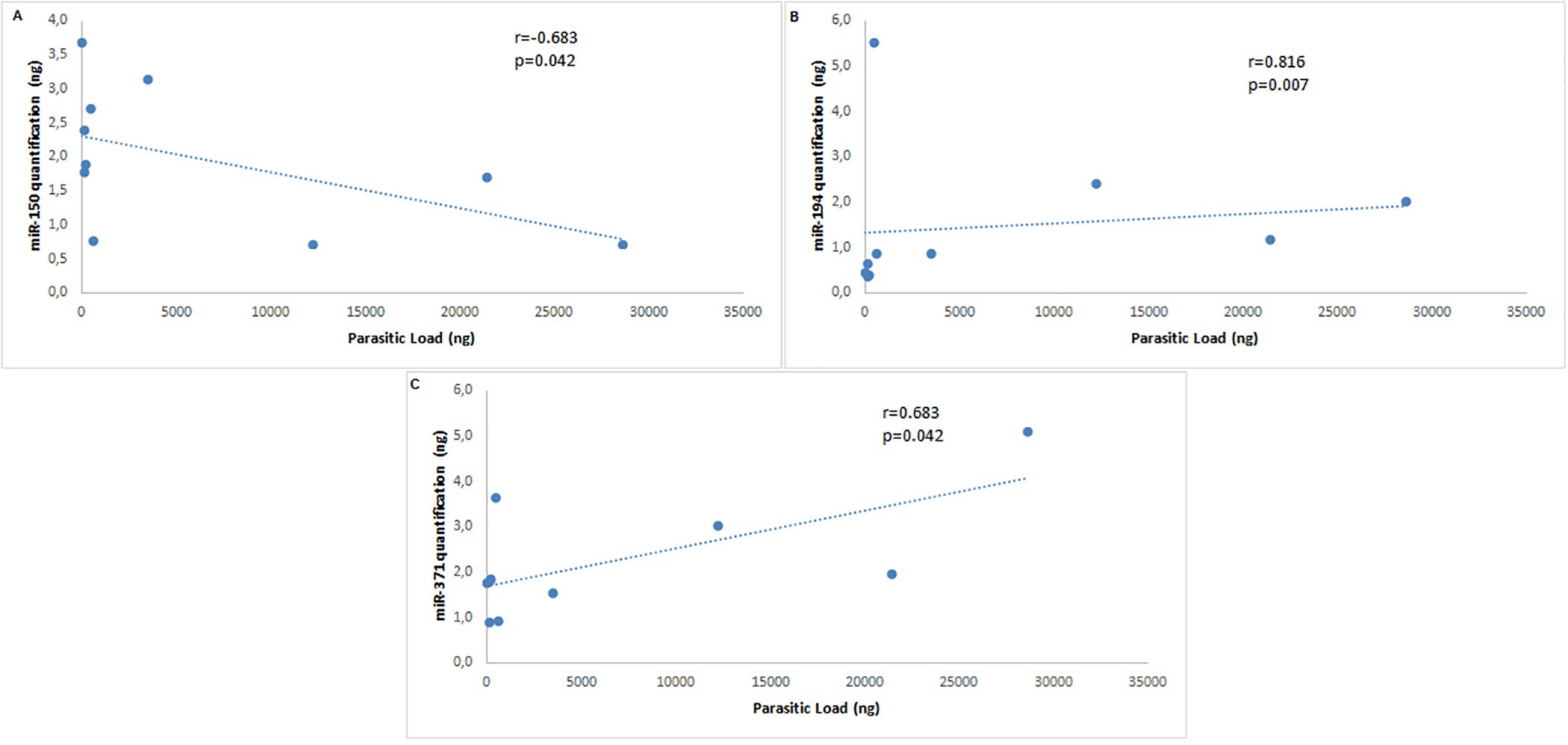
Correlation on miRNAs validated by RT-PCR and parasite load in PBMC of dogs infected by *L*. *infantum*. Data represents a negative correlation of miR-150 (A) and positive correlation of miR-194 (B) and miR-371 (C).

There was a strong positive correlation between parasite load and miR-194 expression (r = 0.816 p = 0.007) and a regular positive correlation between parasite load and miR-371 expression (r = 0.683 p = 0.042) in PBMC from infected dogs compared to healthy dogs. There was a regular negative correlation between parasite load and the expression of miR-150 (r = -0.683 p = 0.042) in these cells. Fig 3 illustrates the correlations between these miRNAs and parasite load.

## Discussion

*Leishmania infantum* was the species observed in dogs with VL in the endemic area studied. Identification of the infecting species is important because differences in miRNA expression by *L*. *donovani* and *L*. *major* have already been observed in infected human dendritic cells *in vitro* [12]. Also, *L*. *amazonensis* has been identified in dogs with visceral leishmaniasis in the nearby city of Bauru-SP, Brazil [20], thus, the determination of *L*. *infantum* in the samples was important to ensure the subsequent results of the research. We demonstrated by microarray that there was altered expression of nine miRNAs in PBMC of animals with CVL compared to healthy animals; the miRNAs miR-21, miR-192, miR-194, miR-371, miR-503, miR-424 and miR-451 were upregulated, and miR-574 and miR-150 were downregulated in CVL samples. Differentially expressed miRNAs in the microarray in the *Canis familiaris* species were also evaluated by real-time PCR. miR-21, miR-150, miR-451, miR-192, miR-194 and miR-371 qPCR results confirmed the microarray data in PBMC. miR-424, miR-574 and miR-503 showed similar patterns to microarray results, but statistical significance was not achieved, likely due to the small sample size (10 infected animals and 5 healthy animals).

The IPA program was efficient in identifying gene targets and demonstrated 63 canonical pathways regulated by these miRNAs, including p53 signaling, antiproliferative role of TOB in T cell signaling, STAT3 pathway, PTEN signaling, death receptor signaling and crosstalk between dendritic cells and natural killer cells involved in immune response. GO analysis were performed for the predicted target genes of the differentially expressed miRNAs in PBMC from dogs with visceral leishmaniasis. GO analysis results showed that the predicted target genes participated in numerous biological processes, suggesting that the six miRNAs could play important roles in regulation of a variety of biological processes. The results for IPA and GO enrichment analyses can therefore provide direction for future research in canine visceral leishmaniasis.

Canonical pathway “crosstalk between dendritic cells and natural killer cells” involved several target genes of differentially expressed miRNAs and their products, between them, TNF-α and IFN-γ are important cytokines in CVL and are related with disease resistance [8], The expression of TNF-α and IFN-γ is downregulated by miR 21 [24–26], which would favor parasitic growth [8].

Canonical pathway analysis also showed relation with apoptosis signaling. Fas and FasL, important molecules in apoptosis, were downregulated by miR-21 [27,28]. Expression levels of Fas and FasL in CD4+ cells from blood and spleen have already been shown to be lower in infected dogs when compared to healthy dogs [29], and the soluble levels Fas (sFas) from spleen leukocytes were lower in dogs with VL compared to controls [30]. Thus, miRNAs are acting in apoptosis pathway that occurs in CVL.

miR-21 showed a significant increase in PBMC of animals with CVL (3.7 fold change). This miRNA is involved in immune regulation, negatively regulating the activation of T lymphocytes [31]. Also, it shows a negative correlation with IL-6 and TNF-α production [32]. TNF-α is associated with resistance in CVL, suggesting that the higher levels of miR-21 may be decreasing TNF-α, which in turn could increase parasite load. In addition, miR-21 negatively regulates proteins involved with adaptative immune response generation, CD40L [33], NFKB [34], CD69 [31], CCR7 [35] and a decreased of NFKB [36], CD40L [37], CCR7 [38] were previously observed in *Leishmania* infection in mice, thus suggesting that miR-21 could be modulation these immune regulatory proteins in CVL.

Likewise, miR-192 showed a significant increase in PBMC of animals with CVL, and it has been shown to down regulate immune response in human [39]. *Leishmania* infection in canine monocyte-derived macrophages decreased expression of costimulatory B7 molecules, and the co-culture with B7-transfected cells resulted in the restoration of cell proliferation and IFN-γ production by a *Leishmania* specific T-cell line [9]. Therefore, it is tempting to suggest that inhibition of T-cell activation and cytokine production are modulated by the expression of miR-192 in dogs with VL.

On the other hand, miR-150 was decreased in PBMC of dogs with VL and had a regular negative correlation with parasitic load in the blood. miR-150 was first described as a key miRNA in the development of B cells [40]. Knockout mice and mutants for miR-150 had an overproduction of immunoglobulins (Igs) of all classes, due to an increase in the number of B1 cells through increased c-Myb gene expression [41]. In dogs with symptomatic VL, there is an increase in the serum level of immunoglobulins of all classes (IgG, IgA IgE, and IgM) [42] and regulatory B cells from *L*. *infantum*–infected dogs co-cultured with enriched T cells, were sufficient to suppress T cell function through IL-10 and PD1 [43]. It is possible that miR-150 is acting in hypergammaglobulin, and in the development of regulatory B-cells, by increasing the parasite burden due to T-cell suppression in CVL.

Increased parasite load associated with decreased miR-150 could also act in other effector pathways of anti-parasitic immunity. Mutant mice lacking miR-150 have a decrease in the number of mature natural killer (NK) cells, whereas mice with increased expression of this miRNA have enhanced development of NK cells [44]. NK cells may play a role in controlling the burden of *Leishmania* spp. parasites during early stages of infection by their ability to respond rapidly to IFN-γ production, which activates macrophages to kill the parasite. *In vitro*, human NK cells were shown to be directly activated by *Leishmania* promastigotes or their lipophosphoglycan (LPG) to produce IFN-γ [45–47]. Thus, the decrease in NK cells modulated by miR-150 expression could be related to a higher parasite load in the CVL animals.

Unlike miR-150, an increase in miR-194 expression was observed in PBMC in dogs with VL and a positive and strong correlation with parasite blood load was observed. In human THP-1 monocytes, an increase in miR-194 expression directly decreases the expression of the TRAF6 gene, decreasing the release of the proinflammatory cytokine TNF-α [48]. In dogs with asymptomatic VL there is a high expression of TNF-α in lymph nodes with low parasitism, and this expression is lower in symptomatic dogs with a higher parasite load, suggesting that this cytokine has a protective function in *L*. *infantum* infection [8]. A miR-194 increase in infected dogs could be regulating the secretion of inflammatory cytokines, such as TNF-α, modulating parasite load in these animals.

Moreover, expression of miR-371 is increased in PBMC of dogs with VL and has a positive correlation with parasite load in PBMC. In patients with asthma, miR-371 targets the Runx3 gene and is related to the Th1 and Th2 response balance, and its increased expression is related to a higher Th2 response [49]. In CVL, the response is both Th1 and Th2, but predominantly Th2, with high production of cytokines by these cells, such as IL-10 and TGF-β, and decreased production of Th1-type cytokines [50,51]. Thus, miR-371 could be involved with pathways that regulate Th2 immune response polarization in CVL.

miR-194 showed a strong positive correlation with serum urea of dogs with VL. This miRNA was increased in plasma of rats with renal ischemia-reperfusion injury [52], dogs with VL can present renal failure [53], suggesting that miR-194 could be explored as an early plasma biomarker in renal lesion in dogs with VL.

Further, miR-194 showed a strong negative correlation with hemoglobin concentration and miR-371 showed a strong negative correlation with erythrocyte globular volume. In CVL hemoglobin and globular volume are decreased [54], as observed in our results, and anemia is a common clinical sign observed in complete blood count of dogs with VL [55]. Other miRNAs have been associated with anemia (miR-150-5p, miR-146b-5p and miR-1) [56] and erythroid homeostasis (miR-144 and miR-451) [57], however miR-194 and miR-371 were not associated with anemia, therefore more studies are needed to confirm our results.

Expression of miR-150, miR-194 and miR-371 showed correlation with parasitic load, indicating that target proteins and pathways should be investigated in future studies. Similarly, human macrophages infected with *L*. *donovani* showed increased expression of miR-30A-3p, and transitory transfection with inhibitor, followed by infection by *L*. *donovani*, promotes autophagy and decrease parasitic load in these cells [58]. These results indicate that differential expression of miRNA is dependent on the parasite species and infected host, thus emphasizing the need for studies in the canine model, the most important disease reservoir.

These findings suggest that *L*. *infantum* may modulate miRNAs in naturally infected dogs. We suggest that their role in immune regulation and their correlation to parasite load may help in the identification of therapeutic targets in Canine Visceral Leishmaniasis.

## Supporting information

S1 Fig PCR-RFLP. Restriction fragment length polymorphism (RFLP) analysis of ITS1-PCR fragments amplified from DNA samples, by using Hae III. M: molecular marker (123 bp); La: *Leishmania amazonensis* (IOC / L0575-MHOM / BR / 1967 / PH8); Lb: *Leishmania braziliensis* (IOC / L0566-MHOM / BR / 1975 / M2903); Li: *Leishmania infantum* (IOC / L0575-MHOM / BR / 2002 / LPC-RPV);1–10: samples profile identical to *Leishmania infantum*. The restriction fragment length polymorphism (RFLP) are indicated by arrows.
(TIFF)

S2 Fig Correlation between miRNAs validated by quantitative PCR and complete blood count and biochemical profile of dogs infected by *L*. *infantum*. Data shows a positive strong correlation of miR-194 and Urea (A), negative strong correlation of miR-194 and Hemoglobin (B) and negative strong correlation of miR-371 and Globular Volume (C).
(TIF)

S1 Table Screening of animals. Optical density on ELISA and clinical signs of naturally infected and healthy animals (control group).
(DOCX)

S2 Table Biochemical analysis. Biochemical profile of animals from infected and control group. Abbreviations: ALT (alanine aminotransferase) AST (aspartate aminotransferase) ALP (alkaline phosphatase) GGT (gamma glutamyl transferase).
(DOCX)

S3 Table Complete blood count. Blood cells of both infected and control groups. Abbreviations: RBC (red blood cells) GV (globular volume) MCHC (Mean corpuscular hemoglobin concentration) MCV (Mean corpuscular volume) TPP (total plasma protein).
(DOCX)

S4 Table Differentially expressed canine miRNAs. Differentially expressed miRNAs of *Canis familiaris* species and respective fold change and p value.
(DOCX)

S5 Table Validation of miRNA differential expression in CVL by real time PCR. Values of efficiency and R^2^ on real time PCR to validate differentially expressed miRNAs.
(DOCX)

S6 Table Ingenuity Canonical Pathways. Canonical pathways predicted for the differentially regulated miRNAs in CVL.
(DOCX)

S7 Table Top 20 GO Biological Process for the targets of differentially expressed miRNAs in CVL. (DOCX)

S8 Table Top 20 GO Cellular Component for the targets of differentially expressed miRNAs in CVL. (DOCX)

S9 Table Top 20 GO Molecular Function for the targets of differentially expressed miRNAs in CVL. (DOCX)
