# Supplementary figures and images for: Relationship of peripheral blood mononuclear cells miRNA expression and parasitic load in canine visceral leishmaniasis
Source: PLoS One. 2018 Dec 5;13(12):e0206876. doi: 10.1371/journal.pone.0206876 (PMC6281177; doi:10.1371/journal.pone.0206876)

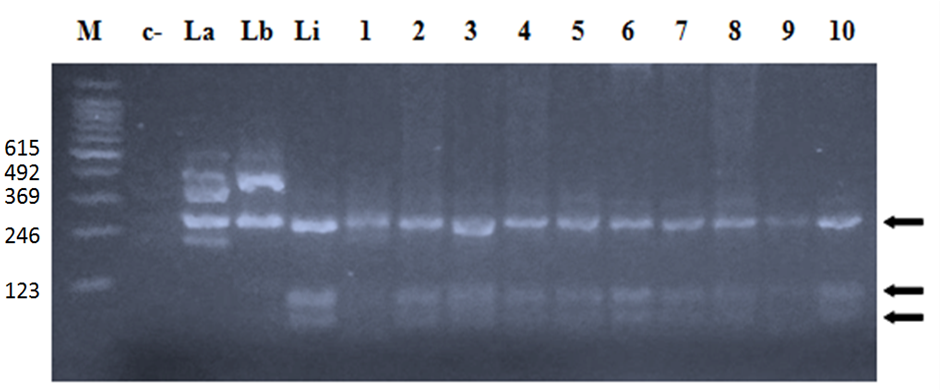

Supplement: S1 Fig — Restriction fragment length polymorphism (RFLP) analysis of ITS1-PCR fragments amplified from DNA samples, by using Hae III. M: molecular marker (123 bp); La: Leishmania amazonensis (IOC / L0575-MHOM / BR / 1967 / PH8); Lb: Leishmania braziliensis (IOC / L0566-MHOM / BR / 1975 / M2903); Li: Leishmania infantum (IOC / L0575-MHOM / BR / 2002 / LPC-RPV);1–10: samples profile identical to Leishmania infantum. The restriction fragment length polymorphism (RFLP) are indicated by arrows. (TIFF) [file pone.0206876.s001.tiff]

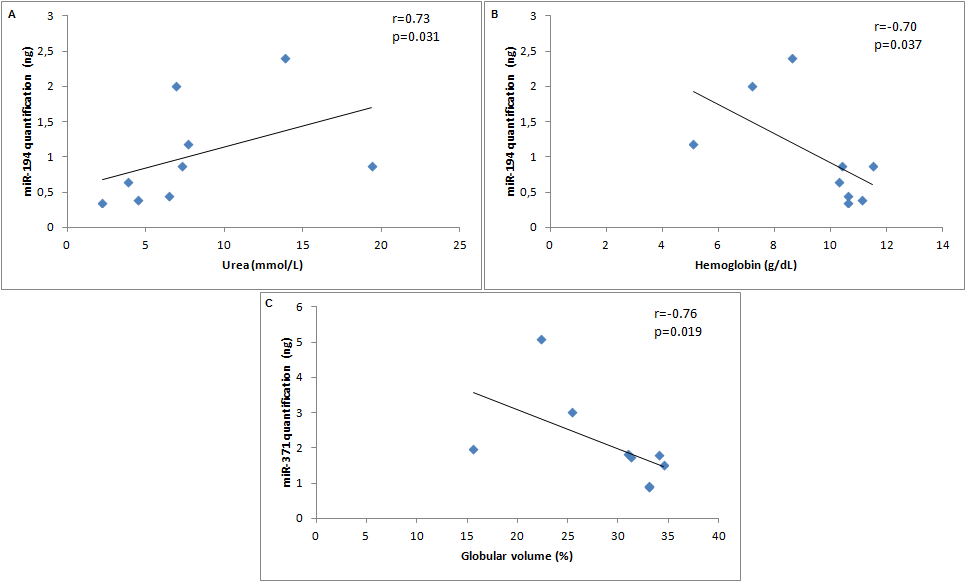

Supplement: S2 Fig — Data shows a positive strong correlation of miR-194 and Urea (A), negative strong correlation of miR-194 and Hemoglobin (B) and negative strong correlation of miR-371 and Globular Volume (C). (TIF) [file pone.0206876.s002.tif]
