# Supplementary material for: Relationship of peripheral blood mononuclear cells miRNA expression and parasitic load in canine visceral leishmaniasis
Source: PLoS One. 2018 Dec 5;13(12):e0206876. doi: 10.1371/journal.pone.0206876 (PMC6281177; doi:10.1371/journal.pone.0206876)
Supplement: S1 Table — Optical density on ELISA and clinical signs of naturally infected and healthy animals (control group). (DOCX) [file pone.0206876.s003.docx]

**S1 Table.** **Screening of animals.**

| **Animal** | **ELISA** | **Clinical Signs** |
| --- | --- | --- |
| Infected 1 | 1.333 | Onychogryphosis, skin lesions, alopecia, ear lesions, anemia, hepatosplenomegaly |
| Infected 2 | 0.511 | Onychogryphosis, cachexia, skin lesions, alopecia, ear lesions, anemia, hepatosplenomegaly |
| Infected 3 | 0.974 | Onychogryphosis, cachexia, ear lesions, anemia |
| Infected 4 | 1.283 | Onychogryphosis, alopecia, ear lesions, anemia |
| Infected 5 | 0.356 | Lymphadenopathy, onychogryphosis, skin lesions, hepatosplenomegaly |
| Infected 6 | 1.323 | Onychogryphosis, skin lesions, ear lesions, anemia |
| Infected 7 | 1.315 | Onychogryphosis, cachexia, skin lesions, ear lesions, anemia |
| Infected 8 | 0.907 | Onychogryphosis, cachexia, ear lesions, anemia, hepatosplenomegaly |
| Infected 9 | 1.089 | Lymphadenopathy, onychogryphosis, skin lesions, alopecia, ear lesions, anemia, hepatosplenomegaly |
| Infected 10 | 0.711 | Onychogryphosis, alopecia, ear lesions, anemia |
| Control 1 | 0.026 | No clinical sings |
| Control 2 | 0.032 | No clinical sings |
| Control 3 | 0.028 | No clinical sings |
| Control 4 | 0.049 | No clinical sings |
| Control 5 | 0.055 | No clinical sings |

Optical density on ELISA and clinical signs of naturally infected and healthy animals (control group).
