# Supplementary material for: Relationship of peripheral blood mononuclear cells miRNA expression and parasitic load in canine visceral leishmaniasis
Source: PLoS One. 2018 Dec 5;13(12):e0206876. doi: 10.1371/journal.pone.0206876 (PMC6281177; doi:10.1371/journal.pone.0206876)
Supplement: S2 Table — Biochemical profile of animals from infected and control group. Abbreviations: ALT (alanine aminotransferase) AST (aspartate aminotransferase) ALP (alkaline phosphatase) GGT (gamma glutamyl transferase). (DOCX) [file pone.0206876.s004.docx]

**S2 Table**. **Biochemical analysis.**

| Animal | Albumin | Uric Acid | ALT | AST | Creatinine | ALP | Total protein | Urea | GGT |
| --- | --- | --- | --- | --- | --- | --- | --- | --- | --- |
| Reference values | 26 – 33 g/L | 0 – 2 mg/dL | 21 – 102 UI/L | 23 – 66 UI/L | 0.5 - 1.5 mg/dL | 20 – 156 UI/L | 54 – 71 g/L | 1.67-8.33 mmol/L | 1.2 - 6.4 UI/L |
| Inf 1 | 8.18 | 0.29 | 22.02 | 21.45 | 1.08 | 34.38 | 45.16 | 19.42 | 0.94 |
| Inf 2 | 14.01 | 0.63 | 32.44 | 93.56 | 0.73 | 55.43 | 89.2 | 4.54 | 2.3 |
| Inf 3 | 14.65 | 0.28 | 29.97 | 31.89 | 1.24 | 92.16 | 65.33 | 13.89 | 5 |
| Inf 4 | 10.36 | 0.83 | 25.08 | 34.49 | 0.74 | 121.25 | 54.81 | 7.33 | 3.24 |
| Inf 5 | 25.57 | 0.44 | 15.42 | 30.12 | 0.84 | 42.96 | 62.42 | 6.46 | 1.93 |
| Inf 6 | 14.77 | 0.84 | 13.28 | 29.08 | 0.53 | 50.18 | 79.65 | 5.68 | 2.2 |
| Inf 7 | 10.34 | 0.93 | 17.41 | 25.08 | 0.64 | 36.15 | 68.44 | 3.87 | 1.36 |
| Inf 8 | 5.45 | 0.21 | 9.89 | 14.39 | 0.52 | 83.52 | 41.5 | 2.25 | 1.02 |
| Inf 9 | 13.09 | 0.45 | 21.39 | 44.78 | 0.7 | 49.41 | 93.95 | 6.94 | 1.5 |
| Inf 10 | 10.21 | 0.58 | 84.27 | 21.45 | 0.58 | 60.09 | 43.22 | 7.67 | 1.58 |
| Ct 1 | 32.87 | 1.31 | 71.86 | 20.83 | 1.1 | 30.26 | 59.37 | 8.46 | 2.91 |
| Ct 2 | 29.77 | 2 | 57.91 | 21.2 | 0.68 | 48.77 | 52.32 | 5.98 | 1.15 |
| Ct 3 | 31.44 | 1.32 | 32.71 | 55.87 | 0.91 | 143.13 | 60.67 | 5.12 | 1.29 |
| Ct 4 | 34.03 | 2.18 | 34.3 | 24.73 | 4.36 | 41.49 | 56.87 | 6.43 | 2.62 |
| Ct 5 | 31.55 | 1.53 | 69.67 | 38.67 | 1.31 | 98.21 | 55.34 | 6.35 | 4.22 |

Biochemical profile of animals from infected and control group. Abbreviations: ALT (alanine aminotransferase) AST (aspartate aminotransferase) ALP (alkaline phosphatase) GGT (gamma glutamyl transferase).
