# Supplementary material for: Relationship of peripheral blood mononuclear cells miRNA expression and parasitic load in canine visceral leishmaniasis
Source: PLoS One. 2018 Dec 5;13(12):e0206876. doi: 10.1371/journal.pone.0206876 (PMC6281177; doi:10.1371/journal.pone.0206876)
Supplement: S3 Table — Blood cells of both infected and control groups. Abbreviations: RBC (red blood cells) GV (globular volume) MCHC (Mean corpuscular hemoglobin concentration) MCV (Mean corpuscular volume) TPP (total plasma protein). (DOCX) [file pone.0206876.s005.docx]

**S3 Table**. **Complete blood count.**

| Animal | RBC | GV | Hemoglobin | MCV | MCHC | Leukocytes | Neutrophils | Lymphocytes | Monocytes | Eosinophils | Basophils | Platelets | TPP |
| --- | --- | --- | --- | --- | --- | --- | --- | --- | --- | --- | --- | --- | --- |
| Reference value | 5.5 - 8.5 x10^6^/µL | 37 – 55 % | 12—18 g/dL | 60 – 77 fL | 32 – 36 % | 6 – 17 x10³/ µL | 3.000 - 11.000/µL | 1.000 - 4.800/µL | 150 - 1.350/µL | 150 - 1.250/µL | Rares/µL | 160 – 430x10³/µL | 6.0 - 8.0 g/dL |
| Inf 1 | 5.05 | 33 | 11.5 | 65.35 | 34.85 | 12.5 | 9.500 | 1.375 | 1.125 | 500 | 0 | 420 | 9 |
| Inf 2 | 4.91 | 31 | 11.1 | 63.14 | 35.81 | 11.8 | 10.738 | 708 | 354 | 0 | 0 | 60 | 10.9 |
| Inf 3 | 3.41 | 25.4 | 8.6 | 74.5 | 25.2 | 10.2 | 7.752 | 1.836 | 510 | 102 | 0 | 82 | 9 |
| Inf 4 | 5.27 | 34.5 | 10.4 | 65.6 | 30.1 | 6.3 | 4.158 | 1.071 | 756 | 63 | 0 | 159 | 6.8 |
| Inf 5 | 4.24 | 31.3 | 10.6 | 74 | 33.8 | 14.8 | 6.808 | 5.032 | 2.220 | 740 | 0 | 168 | 11 |
| Inf 6 | 4.93 | 31.2 | 10.3 | 63.4 | 33 | 14.9 | 9.238 | 3.427 | 1.043 | 1.192 | 0 | 277 | 10.2 |
| Inf 7 | 4.23 | 33 | 10.3 | 63.5 | 33.1 | 12.5 | 10.000 | 1.750 | 375 | 375 | 0 | 331 | 8 |
| Inf 8 | 5.28 | 34 | 10.6 | 60.2 | 32.4 | 9.8 | 6.272 | 2.156 | 392 | 980 | 0 | 145 | 9 |
| Inf 9 | 4.97 | 22.3 | 7.2 | 44.9 | 32.2 | 8.7 | 6.264 | 2.001 | 174 | 261 | 0 | 95 | 9.2 |
| Inf 10 | 2.57 | 15.6 | 5.1 | 60.9 | 32.6 | 23.9 | 20.076 | 2.868 | 478 | 478 | 0 | 165 | 9 |
| Ct 1 | 8.26 | 55 | 18 | 66.58 | 32.72 | 10.2 | 6.324 | 3.468 | 102 | 306 | 0 | 341 | 5.2 |
| Ct 2 | 7.21 | 53.8 | 18.8 | 74.7 | 34.9 | 8.8 | 6.160 | 1.408 | 880 | 352 | 0 | 296 | 6.2 |
| Ct 3 | 5.32 | 41.2 | 12.9 | 77.5 | 31.3 | 13.1 | 6.419 | 6.668 | 174 | 261 | 0 | 341 | 5.2 |
| Ct 4 | 7.43 | 56.7 | 20.4 | 76.4 | 35.9 | 6.4 | 4.480 | 1.024 | 512 | 384 | 0 | 211 | 6 |
| Ct 5 | 7.96 | 59.9 | 20.8 | 75.3 | 34.7 | 10.6 | 7.844 | 1.060 | 424 | 1.250 | 0 | 228 | 7 |

Blood cells of both infected and control groups. Abbreviations: RBC (red blood cells) GV (globular volume) MCHC (Mean corpuscular hemoglobin concentration) MCV (Mean corpuscular volume) TPP (total plasma protein).
