# Supplementary material for: Relationship of peripheral blood mononuclear cells miRNA expression and parasitic load in canine visceral leishmaniasis
Source: PLoS One. 2018 Dec 5;13(12):e0206876. doi: 10.1371/journal.pone.0206876 (PMC6281177; doi:10.1371/journal.pone.0206876)
Supplement: S4 Table — Differentially expressed miRNAs of Canis familiaris species and respective fold change and p value. (DOCX) [file pone.0206876.s006.docx]

**S4 Table. Differentially expressed canine miRNAs.**

| miRNA | Fold change | P value |
| --- | --- | --- |
| cfa-miR-150 | -2.23 | 0.0043 |
| cfa-miR-574 | -2.67 | 0.0086 |
| cfa-miR-21 | 3.7 | 0.0052 |
| cfa-miR-424 | 3.19 | 0.0081 |
| cfa-miR-192 | 2.78 | 0.0160 |
| cfa-miR-194 | 3.08 | 0.0177 |
| cfa-miR-451 | 3.05 | 0.0218 |
| cfa-miR-503 | 2.01 | 0.0230 |
| cfa-miR-371 | 2.89 | 0.0288 |

Differentially expressed miRNAs of *Canis familiaris* species and respective fold change and p value.
