# Supplementary material for: Relationship of peripheral blood mononuclear cells miRNA expression and parasitic load in canine visceral leishmaniasis
Source: PLoS One. 2018 Dec 5;13(12):e0206876. doi: 10.1371/journal.pone.0206876 (PMC6281177; doi:10.1371/journal.pone.0206876)
Supplement: S5 Table — Values of efficiency and R2 on real time PCR to validate differentially expressed miRNAs. (DOCX) [file pone.0206876.s007.docx]

**S5 Table. Validation of miRNA differential expression in CVL by real time PCR**

| miRNA | Efficiency | R² |
| --- | --- | --- |
| cfa-miR-150 | 1.02 | 0.947 |
| cfa-miR-574 | 0.95 | 0.998 |
| cfa-miR-21 | 1.13 | 0.985 |
| cfa-miR-424 | 1.42 | 0.967 |
| cfa-miR-192 | 0.84 | 0.952 |
| cfa-miR-194 | 0.97 | 0.995 |
| cfa-miR-451 | 1.09 | 0.989 |
| cfa-miR-503 | 1.19 | 0.979 |
| cfa-miR-371 | 1.10 | 0.933 |

Values of efficiency and R² on real time PCR to validate differentially expressed miRNAs.
