# Supplementary material for: Relationship of peripheral blood mononuclear cells miRNA expression and parasitic load in canine visceral leishmaniasis
Source: PLoS One. 2018 Dec 5;13(12):e0206876. doi: 10.1371/journal.pone.0206876 (PMC6281177; doi:10.1371/journal.pone.0206876)
Supplement: S6 Table — Canonical pathways predicted for the differentially regulated miRNAs in CVL. (DOCX) [file pone.0206876.s008.docx]

**S6 Table. Ingenuity Canonical Pathways.**

| **Ingenuity Canonical Pathways** | P value | miRNAs in the Pathway |
| --- | --- | --- |
| Aryl Hydrocarbon Receptor Signaling | 0.00014 | miR-150, miR-192, miR-194, miR-21 |
| p53 Signaling | 0.00015 | miR-150, miR-192, miR-194, miR-21 |
| Tumoricidal Function of Hepatic Natural Killer Cells | 0.00030 | miR-150, miR-192, miR-194, miR-21 |
| Osteoarthritis Pathway | 0.00068 | miR-150, miR-192, miR-194, miR-21 |
| Hepatic Fibrosis / Hepatic Stellate Cell Activation | 0.00098 | miR-150, miR-192, miR-194, miR-21 |
| Crosstalk between Dendritic Cells and Natural Killer Cells | 0.00141 | miR-150, miR-192, miR-194, miR-21 |
| dTMP De Novo Biosynthesis | 0.00182 | miR-192 |
| Cell Cycle: G1/S Checkpoint Regulation | 0.00186 | miR-150, miR-192, miR-194, miR-21 |
| STAT3 Pathway | 0.00347 | miR-192, miR-194, miR-21 |
| Human Embryonic Stem Cell Pluripotency | 0.00363 | miR-150, miR-192, miR-194, miR-21 |
| Estrogen-mediated S-phase Entry | 0.00417 | miR-150, miR-192, miR-194, miR-21 |
| Actin Cytoskeleton Signaling | 0.00427 | miR-150, miR-192, miR-194, miR-21 |
| Type I Diabetes Mellitus Signaling | 0.00427 | miR-150, miR-192, miR-194, miR-21 |
| Cyclins and Cell Cycle Regulation | 0.00437 | miR-150, miR-192, miR-194, miR-21 |
| Regulation of the Epithelial-Mesenchymal Transition Pathway | 0.00468 | miR-150, miR-192, miR-194, miR-21 |
| Antiproliferative Role of TOB in T Cell Signaling | 0.00525 | miR-150, miR-192, miR-194, miR-21 |
| Molecular Mechanisms of Cancer | 0.00603 | miR-150, miR-192, miR-194, miR-21 |
| PTEN Signaling | 0.00603 | miR-150, miR-192, miR-194, miR-21 |
| Small Cell Lung Cancer Signaling | 0.00631 | miR-150, miR-192, miR-194, miR-21 |
| Death Receptor Signaling | 0.00912 | miR-150, miR-192, miR-194, miR-21 |
| Cytotoxic T Lymphocyte-mediated Apoptosis of Target Cells | 0.00933 | miR-150, miR-192, miR-194, miR-21 |
| Induction of Apoptosis by HIV1 | 0.00977 | miR-150, miR-192, miR-21 |
| Sumoylation Pathway | 0.01047 | miR-150, miR-192, miR-194, miR-21 |
| Adipogenesis pathway | 0.01047 | miR-150, miR-192, miR-194, miR-21 |
| Chronic Myeloid Leukemia Signaling | 0.01445 | miR-150, miR-192, miR-194, miR-21 |
| Hereditary Breast Cancer Signaling | 0.01445 | miR-150, miR-192, miR-194, miR-21 |
| Ovarian Cancer Signaling | 0.01445 | miR-150, miR-192, miR-194, miR-21 |
| Myc Mediated Apoptosis Signaling | 0.01549 | miR-150, miR-192, miR-194, miR-21 |
| MSP-RON Signaling Pathway | 0.01738 | miR-150, miR-192, miR-194, miR-21 |
| T Helper Cell Differentiation | 0.01820 | miR-150, miR-192, miR-194, miR-21 |
| ILK Signaling | 0.01862 | miR-150, miR-192, miR-194, miR-21 |
| Glioma Signaling | 0.01905 | miR-150, miR-192, miR-194, miR-21 |
| Neurotrophin/TRK Signaling | 0.02042 | miR-150, miR-192, miR-194, miR-21 |
| BMP signaling pathway | 0.02042 | miR-150, miR-192, miR-194, miR-21 |
| Colorectal Cancer Metastasis Signaling | 0.02138 | miR-150, miR-192, miR-194, miR-21 |
| Glioblastoma Multiforme Signaling | 0.02344 | miR-150, miR-192, miR-194, miR-21 |
| p38 MAPK Signaling | 0.02399 | miR-150, miR-192, miR-194, miR-21 |
| ATM Signaling | 0.02455 | miR-150, miR-192, miR-21 |
| Differential Regulation of Cytokine Production in Macrophages and T Helper Cells by IL-17A and IL-17F | 0.02455 | miR-150, miR-194, miR-21 |
| S-methyl-5'-thioadenosine Degradation II | 0.02754 | miR-21 |
| JAK/Stat Signaling | 0.02754 | miR-150, miR-192, miR-194, miR-21 |
| Role of Tissue Factor in Cancer | 0.02754 | miR-150, miR-192, miR-194, miR-21 |
| Histamine Degradation | 0.02754 | miR-194, miR-21 |
| Graft-versus-Host Disease Signaling | 0.02818 | miR-150, miR-192, miR-194, miR-21 |
| RhoA Signaling | 0.02818 | miR-150, miR-192, miR-194, miR-21 |
| Cell Cycle: G2/M DNA Damage Checkpoint Regulation | 0.02951 | miR-150, miR-192, miR-194, miR-21 |
| Acute Phase Response Signaling | 0.03020 | miR-150, miR-192, miR-194, miR-21 |
| Cardiomyocyte Differentiation via BMP Receptors | 0.03020 | miR-192, miR-194, miR-21 |
| Type II Diabetes Mellitus Signaling | 0.03162 | miR-150, miR-192, miR-194, miR-21 |
| IL-6 Signaling | 0.03162 | miR-150, miR-192, miR-194, miR-21 |
| PEDF Signaling | 0.03162 | miR-150, miR-192, miR-194, miR-21 |
| TGF-β Signaling | 0.03162 | miR-150, miR-192, miR-194, miR-21 |
| Bladder Cancer Signaling | 0.03311 | miR-150 , miR-192, miR-194, miR-21 |
| Altered T Cell and B Cell Signaling in Rheumatoid Arthritis | 0.03548 | miR-150, miR-192, miR-194, miR-21 |
| Apoptosis Signaling | 0.03548 | miR-150, miR-192, miR-194, miR-21 |
| Calcium Signaling | 0.03715 | miR-150, miR-192, miR-194, miR-21 |
| Factors Promoting Cardiogenesis in Vertebrates | 0.03802 | miR-150, miR-192, miR-194, miR-21 |
| Differential Regulation of Cytokine Production in Intestinal Epithelial Cells by IL-17A and IL-17F | 0.03890 | miR-150, miR-194, miR-21 |
| Pyrimidine Deoxyribonucleotides De Novo Biosynthesis I | 0.03890 | miR-192, miR-194, miR-21 |
| Melanoma Signaling | 0.03981 | miR-150, miR-192, miR-194, miR-21 |
| Tyrosine Biosynthesis IV | 0.04074 | miR-194 |
| Th1 and Th2 Activation Pathway | 0.04266 | miR-150, miR-192, miR-194, miR-21 |
| RAR Activation | 0.04786 | miR-150, miR-192, miR-194, miR-21 |

Canonical pathways predicted for the differentially regulated miRNAs in CVL.
