# Supplementary material for: Relationship of peripheral blood mononuclear cells miRNA expression and parasitic load in canine visceral leishmaniasis
Source: PLoS One. 2018 Dec 5;13(12):e0206876. doi: 10.1371/journal.pone.0206876 (PMC6281177; doi:10.1371/journal.pone.0206876)
Supplement: S7 Table — (DOCX) [file pone.0206876.s009.docx]

**S7 Table. Top 20 GO Biological Process for the targets of differentially expressed miRNAs in CVL.**

| **Index** | **Name** | **P-value** | **Adjusted p-value** | **Z-score** | **Combined score** |
| --- | --- | --- | --- | --- | --- |
| 1 | positive regulation of protein phosphorylation (GO:0001934) | 1.069e-19 | 1.036e-16 | -1.91 | 83.29 |
| 2 | cytokine-mediated signaling pathway (GO:0019221) | 1.271e-20 | 2.464e-17 | -1.35 | 61.64 |
| 3 | regulation of apoptotic process (GO:0042981) | 1.733e-15 | 8.399e-13 | -1.79 | 60.73 |
| 4 | positive regulation of protein serine/threonine kinase activity (GO:0071902) | 2.741e-12 | 4.088e-10 | -1.98 | 52.64 |
| 5 | positive regulation of intracellular signal transduction (GO:1902533) | 5.108e-14 | 1.415e-11 | -1.60 | 48.87 |
| 6 | positive regulation of cell proliferation (GO:0008284) | 3.438e-14 | 1.333e-11 | -1.56 | 48.32 |
| 7 | transmembrane receptor protein tyrosine kinase signaling pathway (GO:0007169) | 7.670e-13 | 1.487e-10 | -1.73 | 48.23 |
| 8 | positive regulation of vasculature development (GO:1904018) | 2.203e-10 | 2.136e-8 | -2.06 | 45.86 |
| 9 | positive regulation of MAP kinase activity (GO:0043406) | 1.608e-13 | 3.465e-11 | -1.50 | 44.16 |
| 10 | negative regulation of transcription, DNA-templated (GO:0045892) | 1.867e-11 | 2.129e-9 | -1.74 | 42.97 |
| 11 | positive regulation of transcription, DNA-templated (GO:0045893) | 9.693e-12 | 1.175e-9 | -1.69 | 42.90 |
| 12 | positive regulation of gene expression (GO:0010628) | 5.549e-12 | 7.685e-10 | -1.65 | 42.84 |
| 13 | cellular response to cytokine stimulus (GO:0071345) | 1.815e-16 | 1.173e-13 | -1.17 | 42.33 |
| 14 | regulation of endothelial cell chemotaxis to fibroblast growth factor (GO:2000544) | 0.00001912 | 0.0002923 | -3.52 | 38.27 |
| 15 | activation of protein kinase activity (GO:0032147) | 6.774e-14 | 1.642e-11 | -1.26 | 38.17 |
| 16 | positive regulation of transcription from RNA polymerase II promoter (GO:0045944) | 1.367e-9 | 1.104e-7 | -1.86 | 37.97 |
| 17 | regulation of MAP kinase activity (GO:0043405) | 1.261e-8 | 6.986e-7 | -2.08 | 37.79 |
| 18 | regulation of intracellular signal transduction (GO:1902531) | 8.830e-9 | 5.188e-7 | -1.92 | 35.67 |
| 19 | positive regulation of cell migration (GO:0030335) | 6.738e-9 | 4.214e-7 | -1.87 | 35.27 |
| 20 | positive regulation of extrinsic apoptotic signaling pathway via death domain receptors (GO:1902043) | 0.00001201 | 0.0001993 | -3.09 | 35.03 |
