# Supplementary material for: Relationship of peripheral blood mononuclear cells miRNA expression and parasitic load in canine visceral leishmaniasis
Source: PLoS One. 2018 Dec 5;13(12):e0206876. doi: 10.1371/journal.pone.0206876 (PMC6281177; doi:10.1371/journal.pone.0206876)
Supplement: S8 Table — (DOCX) [file pone.0206876.s010.docx]

**S8 Table. Top 20 GO Cellular Component for the targets of differentially expressed miRNAs in CVL.**

| **Index** | **Name** | **P-value** | **Adjusted p-value** | **Z-score** | **Combined score** |
| --- | --- | --- | --- | --- | --- |
| 1 | platelet alpha granule lumen (GO:0031093) | 5.294e-7 | 0.00003494 | -1.98 | 28.65 |
| 2 | platelet alpha granule (GO:0031091) | 2.740e-7 | 0.00003494 | -1.64 | 24.76 |
| 3 | death-inducing signaling complex (GO:0031264) | 0.001744 | 0.02302 | -3.30 | 20.94 |
| 4 | filopodium (GO:0030175) | 0.00007266 | 0.003087 | -2.01 | 19.17 |
| 5 | actin-based cell projection (GO:0098858) | 0.0001502 | 0.003966 | -1.92 | 16.92 |
| 6 | serine/threonine protein kinase complex (GO:1902554) | 0.0008083 | 0.01185 | -2.28 | 16.20 |
| 7 | CD40 receptor complex (GO:0035631) | 0.003153 | 0.03784 | -2.72 | 15.66 |
| 8 | RNA polymerase II transcription factor complex (GO:0090575) | 0.0006647 | 0.01097 | -2.06 | 15.06 |
| 9 | secretory granule lumen (GO:0034774) | 0.00009355 | 0.003087 | -1.28 | 11.90 |
| 10 | integral component of plasma membrane (GO:0005887) | 0.0006494 | 0.01097 | -1.60 | 11.77 |
| 11 | membrane raft (GO:0045121) | 0.0002167 | 0.004768 | -1.34 | 11.32 |
| 12 | extrinsic component of external side of plasma membrane (GO:0031232) | 0.05542 | 0.2454 | -3.42 | 9.88 |
| 13 | spanning component of plasma membrane (GO:0044214) | 0.04866 | 0.2454 | -2.96 | 8.94 |
| 14 | microvillus (GO:0005902) | 0.003513 | 0.03865 | -1.55 | 8.78 |
| 15 | clathrin-sculpted gamma-aminobutyric acid transport vesicle membrane (GO:0061202) | 0.06213 | 0.2454 | -3.03 | 8.42 |
| 16 | nuclear chromatin (GO:0000790) | 0.009653 | 0.07480 | -1.75 | 8.11 |
| 17 | clathrin-sculpted gamma-aminobutyric acid transport vesicle (GO:0061200) | 0.06213 | 0.2454 | -2.80 | 7.79 |
| 18 | cyclin/CDK positive transcription elongation factor complex (GO:0008024) | 0.06213 | 0.2454 | -2.79 | 7.75 |
| 19 | SCF ubiquitin ligase complex (GO:0019005) | 0.007021 | 0.06620 | -1.51 | 7.49 |
| 20 | pseudopodium (GO:0031143) | 0.06879 | 0.2454 | -2.67 | 7.14 |
