# Supplementary material for: Relationship of peripheral blood mononuclear cells miRNA expression and parasitic load in canine visceral leishmaniasis
Source: PLoS One. 2018 Dec 5;13(12):e0206876. doi: 10.1371/journal.pone.0206876 (PMC6281177; doi:10.1371/journal.pone.0206876)
Supplement: S9 Table — (DOCX) [file pone.0206876.s011.docx]

**S9 Table. Top 20 GO Molecular Function for the targets of differentially expressed miRNAs in CVL.**

| **Index** | **Name** | **P-value** | **Adjusted p-value** | **Z-score** | **Combined score** |
| --- | --- | --- | --- | --- | --- |
| 1 | phosphatidylinositol kinase activity (GO:0052742) | 8.031e-8 | 0.000001992 | -2.30 | 37.61 |
| 2 | phosphatidylinositol-4,5-bisphosphate 3-kinase activity (GO:0046934) | 3.073e-8 | 0.000001222 | -1.83 | 31.65 |
| 3 | transmembrane receptor protein serine/threonine kinase activity (GO:0004675) | 0.000003129 | 0.00005542 | -2.47 | 31.30 |
| 4 | growth factor receptor binding (GO:0070851) | 3.244e-7 | 0.000007314 | -1.96 | 29.24 |
| 5 | protein serine/threonine kinase activity (GO:0004674) | 1.416e-10 | 3.512e-8 | -1.26 | 28.63 |
| 6 | phosphatidylinositol 3-kinase activity (GO:0035004) | 3.803e-9 | 3.144e-7 | -1.47 | 28.52 |
| 7 | kinase binding (GO:0019900) | 5.054e-8 | 0.000001393 | -1.54 | 25.96 |
| 8 | cytokine activity (GO:0005125) | 1.205e-9 | 1.494e-7 | -1.23 | 25.25 |
| 9 | thioesterase binding (GO:0031996) | 0.00007356 | 0.0008292 | -2.63 | 25.06 |
| 10 | growth factor activity (GO:0008083) | 3.449e-8 | 0.000001222 | -1.45 | 24.94 |
| 11 | transforming growth factor beta binding (GO:0050431) | 0.00001080 | 0.0001576 | -2.17 | 24.77 |
| 12 | 1-phosphatidylinositol-3-kinase activity (GO:0016303) | 2.412e-8 | 0.000001222 | -1.41 | 24.77 |
| 13 | ubiquitin-like protein ligase binding (GO:0044389) | 0.000001490 | 0.00002842 | -1.80 | 24.09 |
| 14 | fibroblast growth factor receptor binding (GO:0005104) | 0.00009513 | 0.0009496 | -2.50 | 23.18 |
| 15 | tumor necrosis factor receptor binding (GO:0005164) | 0.00007356 | 0.0008292 | -2.40 | 22.86 |
| 16 | cytokine receptor binding (GO:0005126) | 0.000006465 | 0.0001002 | -1.82 | 21.74 |
| 17 | phosphatidylinositol bisphosphate kinase activity (GO:0052813) | 4.321e-8 | 0.000001340 | -1.20 | 20.37 |
| 18 | C-C chemokine binding (GO:0019957) | 0.001744 | 0.008161 | -3.17 | 20.12 |
| 19 | activin-activated receptor activity (GO:0017002) | 0.001363 | 0.007041 | -3.00 | 19.83 |
| 20 | transforming growth factor beta-activated receptor activity (GO:0005024) | 0.001027 | 0.005536 | -2.86 | 19.70 |
